# What are the benefits and risks of nutrition policy actions to reduce added sugar consumption? An Australian case study

**DOI:** 10.1017/S1368980022000234

**Published:** 2022-07

**Authors:** Cherie Russell, Phillip Baker, Carley Grimes, Mark Andrew Lawrence

**Affiliations:** 1 School of Exercise and Nutrition Sciences, Deakin University, Geelong 3220, Australia; 2 Institute for Physical Activity and Nutrition, Deakin University, Geelong, Australia

**Keywords:** Added sugar, Non-nutritive sweeteners, Stakeholder interviews, Reductionism, Political priority, Food industry

## Abstract

**Objective:**

This study aimed to critically analyse Australia’s current and proposed policy actions to reduce added sugar consumption. Over-consumption of added sugar is a significant public health nutrition issue. The competing interests, values and beliefs among stakeholders mean they have disparate views regarding which policy actions are preferable to reduce added sugar consumption.

**Design:**

Semi-structured interviews using purposive, snowball sampling and policy mapping. Policy actions were classified by two frameworks: NOURISHING (e.g. behaviour change communication, food environment and food system) and the Orders of Change (e.g. first order: technical adjustments, second order: reforming the system, third order: transforming the system).

**Setting:**

Australia.

**Participants:**

Twenty-two stakeholders from the food industry, food regulation, government, public health groups and academia.

**Results:**

All proposed and existing policy actions targeted the food environment/behaviour change; most were assessed as first-order changes, and reductionist (nutrient specific) in nature. Influences on policy actions included industry power, stakeholder fragmentation, government ideology/political will and public pressure. Few stakeholders considered potential risks of policy actions, particularly of non-nutritive sweetener substitution or opportunity costs for other policies.

**Conclusions:**

Most of Australia’s policy actions to reduce added sugar consumption are reductionist. Preferencing nutrient specific, first-order policy actions could reflect the influence of vested interests, a historically dominant reductionist orientation to nutrition science and policy, and the perceived difficulty of pursuing second- or third-order changes. Pursuing only first-order policy actions could lead to ‘regrettable’ substitutions and creates an opportunity cost for more comprehensive policy aimed at adjusting the broader food system.

The over-consumption of added sugar, defined as all mono- and di-saccharides added to foods by the manufacturer, cook or consumer^(1)^, is a significant global public health nutrition problem. High intakes of added sugar are associated with type-two diabetes, atherosclerosis and dental caries^(2–5)^. This association is particularly pronounced when added sugars are consumed in the form of sugar-sweetened beverages (SSB), which are the most common source of added sugar intakes in Australia^(6)^. The WHO recommends reducing free sugar consumption (consisting of both added sugars and sugars that occur naturally in honey, syrups and fruit) to 10 % of total energy intake^(7)^. Between 42 and 76 % of Australians exceed this recommendation, with adolescents consuming the most added sugar^(8)^.

Many policy actions have been proposed globally to reduce added sugar consumption^(9)^. Policy actions can target different areas of the food system to alleviate nutrition problems: ‘food supply’ policies are cross-sectoral and target upstream economic and social systems; ‘food environment’ policies target the spaces in which food choices are made, and ‘behaviour change communication’ policies provide education and skills to encourage consumers to change their behaviours^(10)^. To date, most policy actions implemented globally to reduce added sugar consumption have targeted the behaviour change communication and food environment domains, as opposed to the food supply^(11)^. Examples include SSB levies, consumer education, front of pack labelling and reformulation targets^(11)^. These policy actions often focus on added sugar in isolation, rather than interventions that influence the social and commercial determinants of health and improve the accessibility, availability and affordability of healthy and sustainable foods.

Reformulation to reduce the added sugar content of packaged foods may be offset with the addition of other substances, including sweet proteins, bulking agents, and most commonly, non-nutritive sweeteners (NNS)^(5)^. NNS are substances with non-nutritive properties^(12)^ which are used to reduce the energy and sugar content of packaged foods while maintaining their palatability^(13)^. The health risks of NNS consumption are contested^(14)^, with industry sponsorship and authors’ financial conflicts of interest influencing the evidence base^(15)^. Clinical trials have demonstrated a reduction in BMI^(16–18)^ and fasting blood glucose^(19,20)^. However, observational studies have reported associations between NNS consumption and weight gain^(21–24)^, changes to the gut microbiome^(25)^ and type-two diabetes^(26,27)^.

The policy-making process in which policy actions are prioritised, developed and implemented is complex and is influenced by power relations within and between regulating bodies, industry and government; technological advances; economic conditions; pressure from consumers and stakeholder worldviews and framing^(28–32)^. In part, due to their vested interests, values and beliefs, different stakeholders often have disparate views regarding which policy actions are preferable to improve global diets, including reducing added sugar consumption^(33,34)^. These worldviews can be distinguished as being either more ‘holistic’ or more ‘reductionist’ in their characterisation of public health problems^(35)^. Stakeholders who subscribe to a holistic worldview of the causes of public health problems generally advocate for policy actions that address the entire food system and its underlying social, commercial and structural drivers^(36)^. Conversely, stakeholders who subscribe to a reductionist worldview of the causes of public health problems generally advocate for policy actions that target adjustments to the nutrient and ingredient composition of food products^(36)^. Despite the influence of differing worldviews towards the use of regulation, little is known about why stakeholders champion certain policies over others and what they perceive as the risks and benefits of particular policy actions.

There are numerous stakeholders that may influence the formulation of policy to reduce added sugar consumption in Australia, rather than policy makers alone^(37)^. This includes The Australia and New Zealand Ministerial Forum on Food Regulation (The Forum) and their advisers, The Food Regulation Steering Committee (FRSC); members of the regulatory body *Food Standards Australia and New Zealand* (FSANZ); the food industry (including growers, millers, manufacturers and industry representative bodies); public health and nutrition organisations; government bodies, and academics. This paper asks: What types of policy actions do stakeholders champion to reduce added sugar intakes, and why? Do stakeholders discern that policy actions targeting added sugar consumption may impact the level of NNS in the food supply? What do stakeholders perceive to be the risks/benefits of different policy actions? As public health policy actions impact the food supply and consequently consumption patterns, understanding how and why these stakeholders promote certain policy actions to reduce added sugar consumption is important to inform future food and nutrition policy debates.

This study aims to critically analyse current and proposed policy actions to reduce added sugar consumption. The study has three objectives: first, to describe Australia’s current and proposed policy actions to reduce added sugar consumption; second, to determine which factors influence the policy preference of governing bodies, and third, to understand what stakeholders perceive as the benefits and harms of different policy actions.

## Methods

This paper presents a case study of nutrition policy actions to reduce added sugar consumption in Australia. Case studies, in which the learnings from the subject under study provide insights to other settings and contexts^(38)^, are a commonly used method to analyse complex, multivariable topics involving human participants^(39)^. We held a relativist ontological position and used an epistemology that embraced subjectivity^(40,41)^. The methods and results of this study have been reported according to the Consolidated Criteria for Reporting Qualitative Research (COREQ) checklist^(42)^.

### Sampling and recruitment

Participants were individuals involved in decision making, or with knowledge and expertise in public health and nutrition policy, including members of national food regulation bodies, food industry groups (growers, millers, manufacturers and industry representative bodies), public health and nutrition organisations, government departments, and academics. Purposive, snowball sampling was used to identify participants, including from submissions to FSANZ consultations regarding NNS and individuals known to the researchers.

### Data collection

This study used semi-structured interviews to gain insights from stakeholders about policy actions to reduce added sugar consumption, and policy mapping of existing policy actions. Interviews are a frequently used method in public health research to explore stakeholder’s specialist knowledge, worldviews and narrative framing^(43,44)^. Semi-structured interviews were conducted by CR, a PhD candidate and trained qualitative nutrition researcher, via Zoom between July and October 2020. Following consent, interviews were recorded and transcribed. Given the politically sensitive nature of the topic, informants are identified by their sector only. Twenty-two participants were included in the study, as described in Table 1. Participants were asked their views on current or future policy actions, factors that influenced policy making in Australia, and whether focusing on added sugar would improve population diets. Transcripts were not returned to participants for comment.

**Table 1:** Description of participants recruited for interviews on sugar policy in Australia

Sector	Informants contacted	Informants declined	Non-respondents	Participants included
Food regulation	10	5	1	4
Food industry	7	0	4	3
Public health organisation	10	3	0	7
Civil servants	8	0	3	5
Academia	3	0	0	3
Total	38	8	10	22

The World Cancer Research Fund’s NOURISHING database was searched by CR to identify existing policy actions to reduce added sugar consumption in Australia^(9)^. This global database provides information on all policy actions to improve public health nutrition, is updated frequently, and is populated through a two-stage review and verification process^(45,46)^. All policy actions recorded in the database for the Australian context were retrieved. Policy actions were discussed among all authors to determine whether they may influence the consumption of added sugar, either directly or indirectly. This information was corroborated and supplemented with data retrieved from the websites of federal and state government departments and statutory agencies. Given the area under research was government legislation and regulation, only policy actions implemented by government or food regulating bodies were included in the mapping analysis. Frameworks, proposals and calls for policy that were not implemented by governments at the time of analysis were not included.

### Data analysis

Thematic analysis was used to categorise key themes. After familiarisation, interviews were coded by CR using an iteratively derived coding schema in NVivo qualitative analysis software (QSR International, Version 12). Themes were discussed and finalised among all authors. All policy actions obtained from the NOURISHING database and government websites were categorised using two frameworks: (i) a modified version of the NOURISHING framework domains: the food supply, the food environment and behaviour change communication^(9)^ and (ii) the Orders of Food Systems Change schema^(47)^. In this schema, solutions to public health nutrition problems are categorised as three orders of change. This includes first-order (technical inefficiencies within the system which require technological adjustments), second-order (operational shortcomings within the system which require structural reforms) and third-order (a broken system that requires systemic transformation) changes (Table 2). For the NOURISHING framework, the ‘food system’ category was modified to depict the ‘food supply’, as this was considered a better representation of the types of policy actions implemented to improve public health nutrition issues.

**Table 2:** The Orders of Food Systems Change schema^(47)^

Criterion	First-order change (adjust)	Second-order change (reform)	Third-order change (transform)
How the problem is framed, and its cause ascribed to the food system	If a problem exists, it is a consequence of technical inefficiencies within the system design	Accepts that there is a problem, and its cause(s) are associated with structural and operational shortcomings within the system	Accepts the problem as a real and present danger and a consequence of a broken system created from flawed social, economic and political values
Process for change	Preserves the established power structure and relationships among actors in the system	Challenges established power relationships shaping components within the system; promotes opportunities for interactions among a diverse range of actors in the system	Promotes change in relationships towards whole-system awareness and identity; promotes examination of the deep structures that sustain the system
Participation of stakeholders	Replicates the established decision-making group and power relationships. Tends to be global in scope	Brings relevant actors (government, civil society, academics and practitioners, producers, food industry) into the problem-solving conversation in ways that enable them to influence the decision-making process	Promotes social inclusion, empowered producers and citizens actively engaged with the food system instead of being passive takers. Tends to be local in scope
Example of the policy approach to bring about food system change	Applies technological innovations to improve the resilience and/or adaptive capacity of components of the food system (e.g. reformulation)	Applies a mechanistic analysis to identify leverage points within the system (different levels of government and/or sectors with responsibilities for system components) to reform their structure and operation (e.g. sugar levies and subsidies for fruits and vegetables)	Applies a systems-level analysis to identify the system’s purpose and power relationships to reorientate its function from being predominantly a component of the industrialised economy to a health, social, environmental and economic resource (e.g. regenerative agriculture)

## Results

### Current policy actions to reduce added sugar consumption

Fifteen examples of implemented policy actions relating to public health nutrition in Australia were retrieved from the NOURISHING database. A further forty-eight were retrieved from the websites of federal and state government departments and statutory agencies. All sixty-three policy actions were determined to have either an explicit or implicit impact on added sugar consumption and thus were included in the analysis. A summary of these policy actions is shown in Table 3.

**Table 3:** Policy actions that implicitly or explicitly impact added sugar consumption in Australia

Order of change^*^	Domain^†^	Type of policy^†^	Policy action^†^	Example/Description	Regulatory status	States/territories engaged
First-order change (Adjust)	Food Environment	*Nutrition label standards and regulation of the use of claims and implied claims on food*	Clearly visible Interpretive labels	Health Star Rating System – Interpretative front of pack labelling developed in collaboration with industry, public health experts and consumer groups calculates a star rating based on nutrient profiling	Voluntary	All (and New Zealand)
			Mandatory nutrients lists on packaged food	Nutrition Information panel – Producers and retailers are required by law to provide a list of the nutrient content of pre-packaged food products (with limited exceptions)	Mandatory	All
			Calorie and nutrient labelling on menus and displays in out-of-home venues	Restaurant chains with ≥ 20 outlets in the state (or seven in ACT), or 50 or more across Australia, must display the kilojoule content of food products on their menu boards. ‘Average adult daily energy intake of 8700 kJ’ must also be featured. Other chains/food outlets can provide this information on a voluntary basis	Voluntary and mandatory	ACT, NSW, SA, QLD, VIC
			Rules on nutrient claims	FSANZ restrictions on nutrient claims – Nutrition, Health and Related Claims Standard 1.2.7 offers rules on the use of nutrition content claims. Although nutrition content claims need to meet certain criteria set out in the standard, there are no generalised nutrition criteria that restrict their use on ‘unhealthy’ food	Mandatory	All (and New Zealand)
			Rules on health claims	FSANZ restrictions on health claims – Nutrition, Health and Related Claims Standard 1.2.7 offers rules for general level (nutrient function) and high level (disease risk reduction) health claims on food labels and in advertisements. Both types of health claims are permitted on food that meet nutrition criteria, defined by the nutrient profiling scoring criterion set out in the standard	Mandatory	All (and New Zealand)
		*Other healthy food standards in public settings*	Standards/guidelines for food available in schools	Guidelines used to assist healthier choices in schools are based on the Australian Dietary Guidelines and Guide to Healthy Eating. Most use a traffic light system: red (limit), amber (choose carefully) and green (best choices). NSW classifies food and drinks as either ‘everyday’ or ‘occasional’. To varying degrees, the guidelines in each jurisdiction cover: the types of products available for sale; product advertising and promotion; use of products for fundraising, rewards, incentives, prizes/giveaways; catering for meetings/events; sponsorships	Voluntary and mandatory	Mandatory – (ACT, WA, SA, NT) Voluntary – (NSW, QLD, VIC, TAS)
				Voluntary school food guidelines ‘National Healthy School Canteens: guidelines for healthy food and drinks supplied in school canteens’ introduced in 2011, based on the Guide to Healthy Eating and the Australian Dietary Guidelines. Food is categorised using the traffic light system. Food in the green category should be actively promoted. Food in the red category should not be sold in school canteens. Implementation of the guidelines is at the discretion of each state/territory	Voluntary	All
				‘Red category’ food is either completely banned in schools or heavily restricted	Mandatory	ACT, NSW, NT, QLD, SA, WA
				Removal of vending machines from public schools	Mandatory	ACT
			Fruit and vegetable initiatives in schools	Crunch and sip fruit and vegetable programme – vegetable and fruit programme promoting the consumption of fruit, vegetables and water during class time – involves students bringing in fruit, vegetables and water from home	Voluntary	WA, NSW, SA, QLD
			Standards/guidelines for food available in other settings	Choose Well, Live Well – Guidelines to promote healthy food and drink choices for remote area camp food services using traffic light labelling	Voluntary	QLD
				‘Food for Sport’ guidelines (includes good sports healthy eating program) – Traffic light labelling guidelines for sporting club canteens	Voluntary	QLD
				‘A Better Choice’ – Healthy food and drink supply strategy for Queensland Health, aims to improve the availability and promotion of healthier food and drinks in Queensland facilities and workplaces – Based on the Australian Dietary Guidelines and uses a traffic light system	Voluntary	QLD
				Healthy Options WA: Food and Nutrition Policy for WA Health Services and Facilities, aims to improve and maintain the health of staff and the broader community by providing health care establishments that support and model good nutrition and healthy eating options – applies to all settings and occasions where food and drinks are available to staff, visitors and outpatients within WA Health. Uses the traffic light system based on nutrient content and alignment with the Australian Dietary Guidelines	Mandatory	WA
			Increased access to water in public areas	In partnership with public venues and the City of Melbourne (including sports facilities, art galleries and stadiums), VicHealth have increased the accessibility to bottle-free water through water fountains and refill stations, and increased stock of reusable water bottles	Mandatory	VIC
				‘Refill Canberra’ sticker – placed on venues where consumers can refill water bottles for free without making a purchase	Voluntary	ACT
		*Use of economic tools to address food affordability and purchase incentives*	Exemption of produce from Goods and Services Tax (GST)	Fruits and vegetables (among other core foods) are exempt from GST to improve their affordability and accessibility	Mandatory	All
		*Restrict food advertising and other forms of commercial promotion*	Restrictions on junk food advertisements	Products defined as junk food, fast food or unhealthy food and drinks by the Australian Dietary Guidelines and Guide to Healthy Eating cannot be advertised on buses and light rails	Mandatory	ACT
		*Improve the quality of the food supply*	Voluntary reformulation targets	Implemented in two waves (first wave July 2020). The first wave has a Na target for twenty-seven food categories, and saturated fat target for five food categories. Reformulation targets apply to 80% of the product category by sales volume for participating businesses, with businesses to show effort towards reformulating the remaining 20% of products	Voluntary	All
	Behaviour Change Communication	*Inform consumers about nutrition through public awareness*	Development and communication of guidelines	Australian Dietary Guidelines and Guide to Healthy Eating (Eat for Health Website) – Food-based dietary guidelines that involve the translation of recommended nutrient intakes or population targets into a recommended balance of food for a healthy diet	N/A	All
				Infant Feeding Guidelines/Australian National Breastfeeding Strategy – Assists health workers in providing consistent advice to the public about breast-feeding and infant feeding The Australian National Breastfeeding Strategy outlines the priorities of Australian governments at all levels to improve the health of infants and young children by protecting, promoting, supporting and monitoring breast-feeding throughout Australia	N/A	All
			Public awareness campaigns	‘Live Lighter’ – Managed by the Heart Foundation in partnership with the Cancer Council; uses a website, social media, advocacy, and radio, print and TV advertisements to encourage people to eat healthily and be physically active to maintain a healthy weight	N/A	WA, VIC, ACT, NT
				‘H_3_0 Challenge’ – A social marketing campaign encouraging Victorians to make a 30-day pledge to replace every sugary drink they would normally drink with water, supported with a website and educational material	N/A	VIC
				‘8700: Find your ideal figure’ – Initiated by the NSW Food Authority, supports the menu labelling initiative in New South Wales and provides information on kilojoules and healthy eating	N/A	NSW
				‘Unpack the Salt’ – Awareness campaign about hidden salt in processed foods launched by VicHealth and the Heart Foundation	N/A	VIC
				‘2 and 5’ – Fruit and vegetable campaign to promote the consumption of fruits and vegetables	N/A	All
				‘Rethink Sugary Drink’ – education campaign to reduce sugary drink consumption	N/A	VIC
				‘Eat Well Tasmania’ – Promotes healthy eating and opportunities to consume seasonally grown, produced and valued added food through social media channels	N/A	TAS
			Information resources	‘Healthy WA’ – Website that provides information and tips for healthy eating	N/A	WA
				‘Better Health Channel’ – website promoting health literacy and healthy living, provides health consumers with trusted, reliable and easy to understand information – quality assured by the Department of Health and Human Services	N/A	VIC
				‘A Guide to Healthy Weight Loss for Adults’ – Brochure that provides information on weight loss, healthy eating and physical activity	N/A	SA
				‘Healthy Living’ – Website that provides information on and weight loss, healthy eating and physical activity, as well as recipes	N/A	SA
				‘Open Your World’ – provides resources, tools and information to support improving well-being by staying healthy, active and connected	N/A	SA
				‘Healthier Choices Canberra’ – Promotes healthy choices with the following: - Website that promotes businesses with nutritious food and beverage options - Healthier Choices Canberra logo can be displayed by outlets that sell healthy options - Instore tips/tricks for consumers to health them choose a healthier option - Online recipes	Voluntary	ACT
				‘Make Healthy Normal’ website – Offers tips, tools and free programmes to support healthy eating and active living, part of the NSW Government’s NSW Healthy Eating and Active Living Strategy	N/A	NSW
				‘Smart Choices’ – Additional resources to accompany traffic light labelling in schools – includes website, fact sheets and tool kits	N/A	QLD
				‘The Healthy Weight Guide’ – information on how to achieve and maintain a healthy weight, based on Australian and international research, developed by the Australian Government	N/A	All
				‘Tucker Talk’ – Online fact sheets for feeding < 5 years	N/A	TAS
				‘Appetite for Life’ – Online resources for people > 65 years, provides information on food safety, eating on a budget, shopping and cooking for one, improving appetite, osteoarthritis and pureeing food	N/A	TAS
				‘Growing Good Habits’ – Website offers tips and advice on a range of issues including maintaining a healthy weight, ideas for physical activity, practical ways to improve nutrition and recipes	N/A	QLD
				‘Start Them Right’- Booklet for carers and parents to guide feeding for <5 years	N/A	TAS
		*Nutrition Advice and counselling*	Nutrition Counselling/programmes	Nutrition Education Materials Online (NEMO) – Online materials for nutrition education, targeted at patients and health professionals	Voluntary	QLD
				‘Get Healthy’ Information and coaching service – Free telephone-based personal health coaching	Voluntary	All
				Adult Healthy Weight Service – regular healthy weight sessions for adults over 25 – sessions provide advice on diet, reading labels and understanding portion size	Voluntary	ACT
				Community Care Nutrition Service – provides support for adults to improve nutrition and health, provided by Accredited Practising Dietitians and Allied Health Care Assistants	Voluntary	ACT
				Diabetes Nutrition Service – provides group and individual appointments for people with diabetes, gestational diabetes, impaired fasting glucose and impaired glucose tolerance	Voluntary	ACT
				Women, Youth and Children Nutrition – provides free nutrition assessment, counselling and advice for children, young people and pregnant women	Voluntary	ACT
			Training for health professionals	The Clinical Practice Guidelines for the management of overweight and obesity – Intended for use by clinicians including general practitioners, primary health care nurses, primary health care professionals and allied health professionals; the Guidelines follow the primary care ‘5As’ framework: ask and assess, advise, assist, arrange. A range of health benefits are promoted in the guidelines including healthy eating plans, increased physical activity and behavioural modification to help patients manage obesity	Voluntary	All
				Talking to Families about Weight Training – Developed for Department of Health WA and NSW Ministry of Health; free online training course for professionals across WA and NSW. Provides information and training for professionals working with children and families in various capacities who may have a role to play in raising the issue of weight with families or encouraging referrals to appropriate services	Voluntary	WA, NSW
		*Give nutrition education and skills*	Child-care and School-based nutrition education	Nutrition education on curricula – Developed by the Australian Curriculum Assessment and Reporting Authority; addresses food and nutrition education in both the Health and Physical Education and Design and Technologies curriculum. Students learn about food production, the benefits of healthy eating and the preparation of healthy foods, as well as how culture and context shape what they eat. States and territories are responsible for implementation	Voluntary	All
				‘Get up and grow’ – Resources to support early childhood education and care settings (centre-based care, family day care and preschools) to implement the healthy eating and physical activity guidelines	Voluntary	All
				‘Live Life Well @ School’ – Assists schools to develop whole school strategies that support physical activity and healthy eating, and improve the teaching of nutrition and physical education	Voluntary	All
				Kitchen Gardens – Provide opportunities for schools to grow and produce healthy food and connect students with healthy food and lifestyles	Voluntary	All
				‘feedAustralia’ – Online menu planning tool to assist early childhood education/care providers to have healthier menus. The tool compares menus with nutritional guidelines and assists services to meet these guidelines	Voluntary	All
				Physical Activity and Nutrition Out of School Hours Care Program (PANOSH) – resources assisting Outside School Hours services to provide healthy/affordable food choices, and to keep children active during afternoons and holidays	Voluntary	QLD
				Move Well Eat Well – promotes healthy eating and physical activity. Award is available to Tasmanian early childhood services and primary schools. Schools and services are supported to meet key criteria which reinforce healthy eating and promote physical activity	Voluntary	TAS
				‘Healthy Schools Achievement Program’ – provides a framework to help schools enhance current health and well-being initiatives to create healthier environments	Voluntary	VIC
				Refresh.ED Nutrition Education – online resource to help teachers introduce food and nutrition in classrooms from kindergarten to year 10	Voluntary	WA
			Workplace health schemes	Healthy Eating Advisory Service – Delivered by Nutrition Australia, helps organisations to put Healthy Choices into practice and provide/promote healthier foods and drink choices. Includes online learning and menu assessment tools and provides information and guidance on planning healthier menus and creating healthier food environments	Voluntary	All
				Healthier Workplace WA – offers free services to all WA workplaces to help them implement a workplace health and well-being programmes that assists workers make positive lifestyle changes, including online and face to face training	Voluntary	WA
			Community-based nutrition education	‘Food sensations’ campaign – Nutrition and cooking initiative offered by Foodbank WA to schools and community groups	Voluntary	WA
				Healthy Eating in Supported Education toolkit – contains practical information about providing nutritious food for supported accommodation residents, adjusting meals for residents with special needs, and how facilities can help residents to make healthier choices	Voluntary	QLD
				‘Family Food Patch’ – promotion of eating well and increased activity through peer education	N/A	TAS
				‘Healthy Eating Advisory Service’ – supports early childhood services, outside school hours care, schools, workplaces, hospitals, sport and recreation centres, tertiary education and parks to provide healthier foods and drinks in their menus	Voluntary	VIC
				‘Healthy. Happier. ‘Campaign – provides information on healthy eating, specifically around increasing fruit and vegetable intake, portion sizes, beverage choices, eating out and physical activity	Voluntary	QLD
				Better Health Program – Delivered by qualified health professionals, includes interactive activities and games to teach the children about healthy eating and physical activity – provided both face to face and online	N/A	WA

ACT, Australian Capital Territory; NSW, New South Wales; NT, Northern Territory; QLD, Queensland; SA, South Australia; TAS, Tasmania; VIC, Victoria; WA, Western Australia; FSANZ, Food Standards Australia and New Zealand.

* From the Orders of Food Systems Change schema^(47)^.

† From the NOURISHING Framework^(9)^.

All policy actions were considered first-order changes and targeted the food environment (*n* 19) and behaviour change communication (*n* 44). No policy actions addressed the food supply. Policy actions fell into eight of the ten policy categories described by the NOURISHING framework (Table 3). No policy actions were found within the categories of setting incentives/rules for healthy retail environments and harnessing food supply chains. Most policy actions were state specific (*n* 44). Though some behaviour change policies could not be classified as either mandatory or voluntary, of those that could, 11/42 policy actions were mandatory.

### Informant perceptions of policy actions to reduced added sugar consumption

**Fig. 1:**
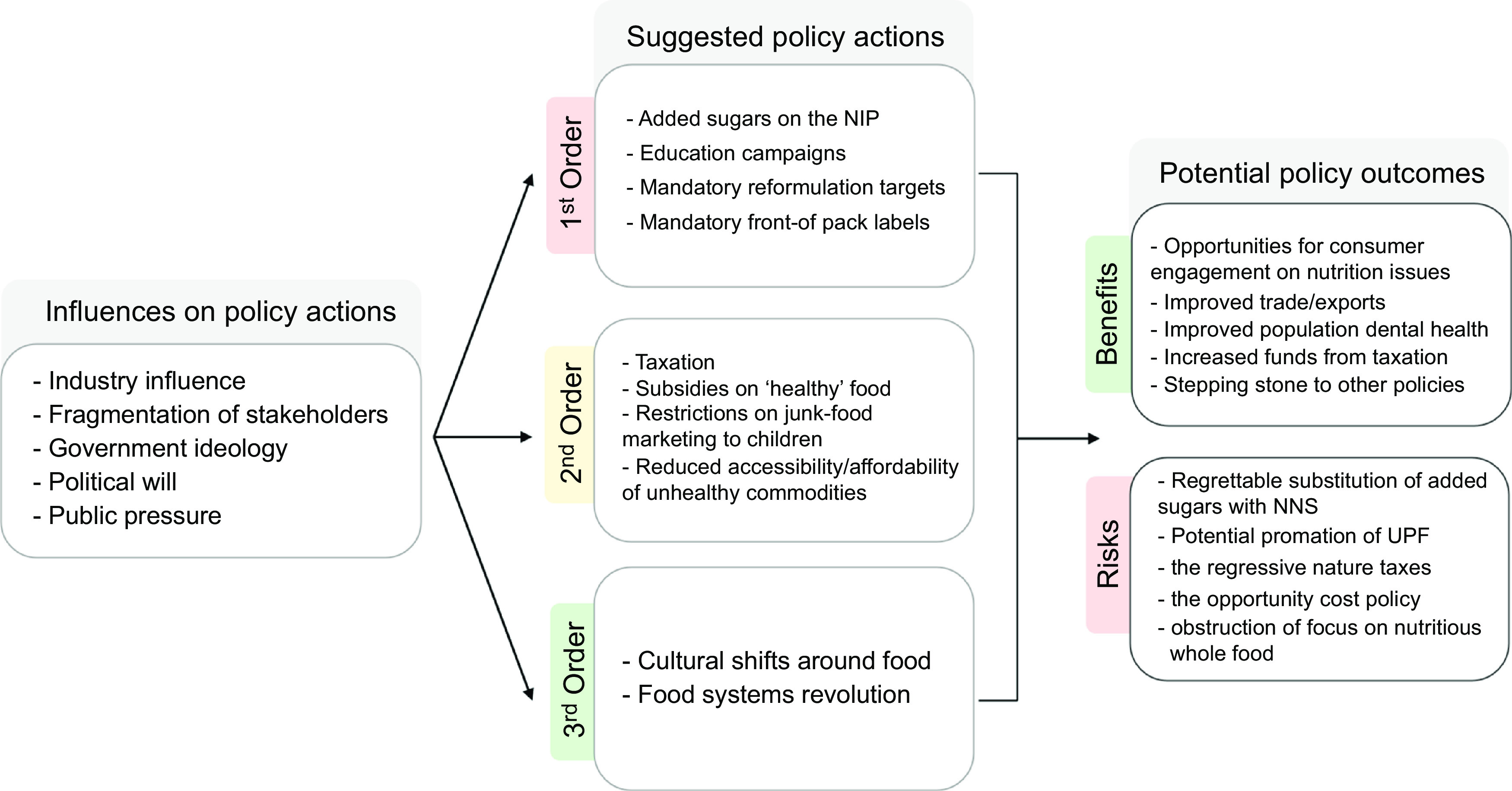
An overview of the informant perceptions of policy actions to reduce added sugar consumption

Three overarching themes emerged from informant interviews (Fig. 1). This included informant perceptions of the influences on policy making to reduce added sugar consumption, potential policy actions that should be implemented to reduce added sugar consumption and the benefits and risks of these policy actions.

### Influences on policy action preferences to reduce added sugar consumption in Australia

Influences on nutrition policy actions to reduce added sugar consumption in Australia identified by informants included industry influence, fragmentation of stakeholders, government ideology and political will, and public pressure.

#### Industry influence

Industry influence was suggested to affect the implementation of added sugar policy actions by all stakeholder groups except those from the food industry. Several academic and public health informants described this influence as detrimental for public health outcomes:

*They’ve got a conflict of interest in creating health policy because they have an obligation to their shareholders to increase and maximize revenue for their products, not to look after the health of people. (Public Health Advocate)*

Coalitions within the industry and their significant financial and human resources, especially in comparison to public health advocates, were raised as mechanisms of industry influence. Other mechanisms of influence suggested by participants included voluntary initiatives to ‘pre-empt’ regulation, relationships with policymakers, having a seat at the policy making table, lobbying government and funding research. The issue of ‘revolving doors’ was also raised by some public health and academic stakeholders, which involves people moving between roles in policy making and industry^(48)^. None of these mechanisms were raised as an influence on policy making by industry informants. Participants suggested that these mechanisms of influence historically resulted in policy decisions that were ‘palatable’ to industry, often resulting in voluntary initiatives rather than regulation:

*I think for us, the challenge was not only that it’s [the Health Stare Rating system] a voluntary system, so it needs to be a system which at least works in some way for the industry. Otherwise, they’re not going to put it on the packet (Public Servant)*

#### Fragmentation of stakeholders

An issue raised by several academic, public health and government informants was the impact of public health sector fragmentation on effective advocacy. Often, this fragmentation was described as a difference between ‘pragmatism’ and ‘idealism’. Some informants suggested that advocacy efforts aimed ‘too low’, with compromises that were too great, while others felt that any policy action was better than nothing:

*From a purist perspective, I absolutely agree that it’s silly to focus on one particular element, such as sugar… we need to be dealing with the diet as a whole… But I also have another view that, I’ve studied a little bit around policy entrepreneurship, policy windows… you have to use those. And so at the moment, sugar is the ‘big bad wolf’, and that’s where the opportunity lies (Public Servant)*

#### Government ideology and political will

‘Political will’ was cited by informants from all stakeholder groups (except for the food industry) as a driver of what policy actions to reduce added sugar consumption are adopted. This included an unwillingness from policy makers to ‘*shake the boat’*, take risks or move away from conservative, institutionalised policies. As stated by one academic: ‘*I don’t really see Australia being an innovator in nutrition policy*’. Others raised the concept of ‘government champions’ who promoted public health policies:

*You can talk about evidence-based policy making – and there’s a place for that, and we need to be ready to inject evidence-based policy in. But the window often gets opened because a political decision maker cares about something (Public Servant)*

Government ideology was raised by several stakeholders as a key influence on both added sugar and nutrition policy generally. It was suggested that the current federal government ‘individualised’ the issue of public health nutrition, placing the onus on individuals to consume healthy diets and reduce added sugar consumption and minimising government responsibility to promote food environments that were not obesogenic:

*Someone who is firmly in that individual court will probably not even entertain environmental interventions, and they’ll describe them as nanny state, over governance, over regulation… people should just control what they put their mouths (Public Servant)*

A theme of preference for minimalistic, or voluntary solutions, was raised by all stakeholder groups:

*Our government’s approach is to do more sort of nonregulatory things. That’s why we have the Healthy Food Partnership or the Health Star Rating… they’re very focused on wanting to have non regulatory actions where industry is engaged in doing things voluntarily and not actually introducing additional regulations (Food Regulator)*

Limited government budgets, favouring economic outcomes over public health, economic reliance on industry and ministerial priorities, particularly in the realm of health during the COVID 19 pandemic, were cited as contributing to the lack of political will to introduce policy actions to reduce added sugar consumption.

#### Public pressure

Community mobility and public pressure were described as imperative to policy change. In particular, sugar was described as a salient issue with the public, easy for consumers to understand and had ‘*policy momentum*’. As stated by a public health advocate, ‘*We can’t ignore the fact that the community is interested… it resonates with people to talk about low sugar, or no added sugar*’. This contrasted with the concept of ultra-processed foods (UPF), which some participants felt could not be easily understood by consumers. Despite this, most participants, regardless of stakeholder group, described increasing the consumption of whole or ‘unpackaged’ foods as the ideal outcome of policy actions.

### Suggested policy actions to reduce added sugar consumption in Australia

#### First-order changes

Potential first-order changes suggested by informants to reduce added sugar consumption included listing added sugars on the nutrition information panel, updating national dietary guidelines, education campaigns, mandatory reformulation targets for added sugar, making the Health Star Rating (HSR system, Australia’s front of pack labelling system) mandatory, and other front-of pack label schemes, including teaspoon labelling of sugar or warning labels.

Changes to the HSR system were the most discussed first-order changes throughout the interviews. Though most participants across all stakeholder categories felt that the HSR system was beneficial, it was also suggested by some academics, public servants and public health advocates that aspects of the system were flawed. This included that it was voluntary, that the nutrient profiling algorithm was lenient on sugar and the suggestion that incorporating added sugar in the HSR algorithm would improve the accuracy of ratings:

*As an overall system it has potential, but there are obviously flaws that we’ve been working to try and fix… in terms of added sugars, there should be much stronger treatment to provide a better indication of the ratings that products should receive (Public Health Advocate)*

Some public health advocates and academics suggested that the system had the potential to mislead consumers regarding the healthiness of a product:

*Health star ratings, [I’m] not a fan of… [they] create a lot of confusion. There is this constraint, consideration, of having been manipulated by industry … They give the impression that some foods that are actually still highly processed might be healthy (Public Health Advocate)*

Reformulation was also a contentious issue among stakeholders, both in terms of mandatory reformulation targets and as a by-product of other policy actions, including the HSR system. Most informants from all stakeholder groups were ‘pro-reformulation’, while some academics and public health advocates raised issues with this approach. Pro-reformulation stakeholders argued it would decrease added sugar intakes without the need for behaviour change from consumers:

*Policies which look at reformulation, so improving packaged food products before the shoppers even pull them off the shelf, has the most scope for reducing inequities… reformulation has the ability to cut across the food supply and take out any kind of decision making that’s required (Public Health Advocate)*

Concerns were raised from some academics regarding reformulation, including describing it as ‘reductionist’: ‘*Are there things we could do to tackle sugar? If you want to go down your reductionist route… that really will not achieve anything*’, and that regardless of added sugar content, the level of processing should be incorporated into policies that may otherwise lead only to nutrient reformulation: ‘*even if I take all the sugar out, it’s still going to be a highly processed, full of additives, heavily marketed food that really, I don’t think anyone would consider healthy*’. Comparatively, some industry stakeholders described the current levels of reformulation as sufficient, with one informant stating, ‘*I don’t think more reformulation needs to be done. I just think that it’s already currently being done*’.

A common theme across informants was that policy actions to encourage reformulation should not be the sole regulatory focus. However, when asked about which policy actions should be implemented to decrease added sugar consumption or improve population health, policies that encouraged reformulation were generally suggested by informants from all stakeholder categories.

#### Second-order changes

Potential second-order changes suggested by informants to reduce added sugar consumption included a SSB tax, subsidies on healthy food, legislated restrictions on junk-food marketing to children and improvements to the food environment, including reducing the accessibility, affordability and desirability of unhealthy commodities for consumers. The ‘obesogenic environment’ was often framed as the cause of obesity by academics and public health advocates, as the current *‘food environment does nothing whatsoever to encourage moderation’ (Academic).* As stated by a public health advocate:

*You need to reduce the availability, change the pricing, change the promotion of these sorts of products… they’re ubiquitous and they’re very cheap and they’re heavily promoted… The very things that create high consumption of these products are the same things we need to stop to reduce consumption (Public Health Advocate)*

A SSB tax was the most recommended second-order change by academics, public health advocates and public servants. Proponents cited the successful use of taxes in other countries, with decreased sugar intakes and increased government revenue. Pro-tax informants suggested that revenue raised from the tax could be used for other health activities, or to help transition sugar farmers to other crops. Industry informants suggested that such a tax was regressive, that the evidence of effectiveness was mixed, that consumers may substitute SSB with other sugary products and that the level of revenue that a tax could raise, and where the funds would be allocated, was uncertain:

*I think there’s been some success with their [UK] sugar levy. And it does drive reformulation. However, what are companies doing? They’re using intense sweeteners… I don’t think that focusing on one nutrient, or one category, is necessarily going to have any great impact. It will raise some money. But will it change obesity levels in the long term? I don’t think so (Industry Representative)*

#### Third-order changes

Only two academic informants recommended third-order changes to reduce added sugar consumption. However, suggestions were non-specific; rather, ‘transformative change’ was highlighted as necessary, as opposed to policy actions that ‘*tinker around the edges*’:

*So the way systems transition is through replacement, rather than reforming things, which is obviously hugely challenging… the system will resist and change and adapt… even though we say complex adaptive systems, this adaptive thing, people seem to just not take any notice of that (Academic)*

### Potential benefits and risks of added sugar policy actions

Potential benefits of policy actions to reduce added sugar raised by informants included increased engagement of consumers and policy makers with nutrition and public health issues, improved international trade and exports, improved population dental health, improved environmental outcomes, increased funds for public health raised through taxation and as a ‘stepping-stone’ to other public health nutrition policies:

*Implementing added sugar policies is one way that we can progress one small part of the nutrition story… if we were to implement a sugary drink health levy… we could refund all that money into other programs or education, subsidising fruit and vegetables (Public Health Advocate)*

Potential unintended risks of policy actions to reduce added sugar consumption suggested by informants included *‘regrettable substitution’* of sugars with NNS, starches or other alternatives; the potential for UPF or foods high in other ‘risk’ nutrients to be promoted as healthy; the regressive nature of some policies (particularly taxes) and the ‘opportunity cost’ of nutrient-specific policy actions.

#### Replacement of added sugar with non-nutritive sweeteners

Informant perceptions of the impact of reformulation of UPF with NNS were mixed in all stakeholder groups. Several participants did not feel that they knew enough about NNS to comment on their efficacy as a sugar substitute. Those who felt that replacing added sugars with NNS was constructive referenced that they were considered safe, as described by an industry informant: ‘*They’re fine to use according to, you know, safety and toxicology studies. They’re fine’*. Several participants felt that while sweeteners were not ideal, they were *‘better than sugar*’. As stated by an industry informant: ‘*Non-nutritive sweeteners can be an effective way of ensuring that consumers don’t consume too much sugar in their diet*’. Other informants suggested that NNS could be used to transition people from added sugar. Some academics, public health advocates and public servants proposed that NNS were an important tool for the food industry, who may not be able to reduce added sugars without their use:

*The challenge with all of this is… what is your end goal… by not limiting sweeteners, you’re providing space for manufacturers to do something, right. If you control sweeteners or restrict them in some way, there’s nowhere for them to go (Academic)*

Some participants distinguished between ‘artificial’ and ‘natural’ NNS, with a preference for natural sweeteners, such as stevia. Concerns were raised generally among academics, public health informants and public servants about the unknown, long-term impacts of NNS consumption, though this was often in tandem with support for using them in reformulation. Academic and public health informants who were not in favour of reformulation with NNS raised concerns about the impact of NNS on gut health, the promotion of unhealthy products containing NNS, impacts on satiety and subsequent energy consumption and that such reformulation may encourage preferences for sweet tastes:

*It also changes people’s taste preferences – that’s a big issue. If people become conditioned to sweeter foods based on these non-nutritive sweeteners as well, it can potentially change what they choose to eat or how much they eat (Public Health Advocate)*

Additional concerns were raised over the efficacy of NNS to improve the healthiness of the food supply overall:

*If the goal is to reduce added sugar consumption, sure, that’s an effective policy. If the goal is to make the products healthier, no. If the goal is to address any of the other problematic dimensions of how the food system in general is unhealthy and dominated by large corporations, etc., No. (Academic)*

#### Opportunity costs of nutrient-specific policies

The potential for nutrient-specific policy actions to divert funding and attention away from more comprehensive policy actions that address dietary patterns, the complexity of obesity or the food system was raised by some academics and public health advocates. As stated by one academic:

*Many of these initiatives, by focusing on changing the products, do very little to address any other issues with the food system, such as market concentration, such as problematic employment practices around the world, such as environmental consequences (Academic)*

Comparatively, participants who felt that focusing on sugar was important often contextualised that ‘it’s not just sugar’, but that focusing on added sugar was ‘*pragmatic’*:

*When you come down to it, the one thing that everybody agrees on is that too much added sugar is not good for you… we just know that the jury has made a decision on sugar. It’s really clear. The evidence is there. (Public Health Advocate)*

## Discussion

The aim of this study was to critically analyse Australia’s current and proposed policy actions to reduce added sugar consumption. The results present a case study for understanding the constraints, enablers and unintended consequences of nutrition policy actions to reduce added sugar consumption.

Though we found sixty-three policy actions (see Table 3) in Australia to reduce added sugar consumption, most were voluntary, and all targeted the food environment and behaviour change as opposed to the food supply. Furthermore, most policy actions were based on nutrient profiling, with exceptions including the dietary guidelines and some behaviour change communication policies. Many of the policies implemented were education campaigns, despite evidence that education has limited efficacy for improving population health when not part of a comprehensive suite of diverse policy actions^(49,50)^. Governments have often preferenced education-based approaches in isolation, which arguably locate responsibility for change within individuals, rather than seeking structural changes to food systems or regulatory actions targeting food environments^(49,50)^. All implemented policy actions were considered ‘first-order changes’ that applied small-scale solutions to perceived technical inefficiencies within the system^(47)^. Likewise, most policy actions proposed by informants, as potential future policies, regardless of stakeholder category, also constituted first-order changes. Industry participants only recommended first-order actions, while third-order actions were only recommended by academics.

Reformulation of food and beverages such as SSB or packaged items, considered UPF, was the most supported policy action across participants, either as an objective or a consequence of other policy actions, such as the HSR system or a tax on SSB. UPF are categorised by the NOVA food processing classification system as industrial formulations which typically contain cosmetic and various other types of additives^(51)^. These products are designed to be hyper-palatable, affordable, convenient and are often marketed intensively^(52)^. The reductionist policy actions implemented and proposed in Australia to reduce added sugar consumption are comparable to those implemented worldwide^(11)^. Such policy actions preserve the established power structure and relationships among actors in the existing system, which can enable the food industry to maintain or expand sales of UPF with altered product compositions or the development of novel products^(47)^. UPF have known adverse health and environmental impacts, including CVD, cancer, type-two diabetes, and all-cause mortality^(53)^, greenhouse gas emissions, deforestation, bio-diversity loss, food waste, increased land clearing and water use^(54,55)^.

The HSR system was the most discussed first-order change to reduce added sugar consumption in this study. Though previous evaluations of the HSR system have shown a positive impact on reformulation, including small changes in energy, Na and fibre^(56–58)^, one study reported no change in added sugar volumes^(59)^, while another found that the number of new products containing added sugar that displayed a HSR was increasing^(60)^. Previous research has demonstrated that the HSR does not distinguish between levels of processing, with three quarters of newly released UPF receiving a HSR of 2·5 stars or higher^(61)^. In comparison to the interpretive nature of the HSR which can inadvertently result in a favourable rating for UPF, the Chilean FOPL system presents mandatory ‘warning’ labels on products high in sugar, salt, saturated fat and energy. The underlying conceptual difference between the two systems has resulted in a 15 % decrease in total sugar in packaged foods and beverages between 2014 (6 months prior to implementation) and 2019^(62)^. Informant support for incorporating the level of processing into the classification of what constitutes a ‘healthy’ food was limited due to concerns of consumer misunderstanding and policy translation difficulties. Level of processing has been included in policy internationally, including as part of the national dietary guidelines of Brazil and Israel^(63,64)^. A SSB tax was the most discussed second-order change by stakeholders, both positively and negatively. To date, forty-five countries have implemented such a tax, with some research suggesting that these taxes decrease sales of SSB^(11,65)^. Their impact on the sales of beverages containing NNS is mixed^(11)^.

Few participants recommended third-order changes to address added sugar intake or dietary imbalance. The focus on added sugar as an isolated nutrition issue, as opposed to a symptom of larger food systems issues, is consistent with reductionist thinking, which remains the dominant paradigm informing public health nutrition policy globally^(66,67)^. Other than an omission of third-order changes from informants, reasons for this lack of support could include the difficulty and complexity of modifying the systems that inform current dietary patterns, the influence of vested interests on the discourse around nutrition issues and the historical influence of reductionism on nutrition science and policy^(66,68,69)^. Despite the lack of support among informants for systemic changes, research suggests that a systems lens should be applied to public health nutrition policy^(69–73)^. Though most policy actions implemented both in Australia and globally have been nutrient focussed, reductionist policy actions are not comprehensive enough to transform global food systems and adequately address the ‘causes of the causes’ of ill health, including the social, commercial, environmental, structural, economic or cultural determinants of health^(69–73)^.

There was a lack of cohesion among informants about whether focussing on added sugar should be prioritised over more comprehensive, but challenging, policy actions. Fragmentation in public health has been documented previously^(74,75)^ and is likely to inhibit political support for action to reduce added sugar intakes^(76)^. Those favouring an expedient approach argued that sugar is currently a salient issue with ‘political priority’ among national populations and international health organisations. Political priority is key for implementing policy actions to improve a public health issue^(76)^. Policy agendas are created when a problem is acknowledged, has ‘viable’ solutions and changes are ‘politically correct’ to make at the time of decision making (known as policy windows)^(77)^. However, pursuing nutrient-specific policy actions may create an opportunity cost for more comprehensive policy aimed at adjusting the broader food system and prioritising human and planetary health. Research in 2020 from I *et al.* ^(78)^ used a case study of the Healthy Food Partnership to demonstrate Australia’s ‘pragmatism’ and inclination to compromise in policy making, suggesting this has led to increasingly narrowed policy responses for ‘wicked’ public health problems.

Few stakeholders had considered the unintended outcomes of policy actions to reduce added sugar consumption, particularly in terms of NNS substitution. Some stakeholders suggested that while this ‘regrettable substitution’ was not ideal, NNS was a better alternative to sugar and may be used to transition people from added sugar. However, when sweetened food is consumed routinely, especially earlier in life, this flavour profile becomes familiar and acceptable and ultimately can inform preferences for sweetened food^(79)^. Evidence suggests that this begins *in utero*, and continues throughout life^(80)^. Overstimulation of sweet taste receptors may limit tolerance for more complex, less sweet tastes, such as fruits and vegetables^(81)^. The promotion of NNS as a ‘lesser of two evils’ ignores their contested health risks, their presence in UPF and the potential ‘health halo’ of NNS containing UPF. While nutrient-specific policy actions that encourage reformulation may decrease levels of added sugar in the food supply, such policies could inadvertently lead to the reformulation and promotion of UPF^(82)^, while also displacing nutritious foods from the diet.

Influence of the food industry on the policy-making process was raised by several informants as a barrier to policy actions to reduce added sugar consumption. This is consistent with previous research identifying the risks and perceptions of commercial power and policy at both the national and international levels^(31,73,74)^. Participants raised the issue of a ‘revolving door’ between government and industry positions. Previous work has recorded the broad extent of this issue in the Australian context, with over a third of registered industry lobbyists previously working in government roles^(48)^. This creates a range of ethical and moral issues in relation to public health, including contributing to the power imbalance between industry and public health advocates, creating ‘industry-friendly networks’, and is theorised to influence nutrition policy setting and contribute to policy inertia^(48)^. Industry influence was described in tandem with a lack of political will and the individualistic approach of Australia’s government to food and nutrition, which has been documented in previous research^(83–85)^. These influences have contributed to a preference for voluntary, industry-led policy actions to address added sugar consumption, rather than legislative action^(31,78)^.

This was the first Australian study to provide insights into stakeholder’s perceptions regarding factors that influence policy actions to reduce added sugar consumption in Australia. Our study included a broad representation of informants from all relevant stakeholder categories and provides insight into how such policies may influence NNS in the food supply and the perceived risks and benefits of these policy actions. This knowledge can be used to inform frameworks for future health promotion and political debates related to food and nutrition policy. Though data from the NOURISHING database are updated frequently and are populated through a two-stage review and verification process, it is limited in terms of capturing the health and sustainability dimensions of food systems, such as by detailing optimum governance arrangements^(86)^. Perspectives of stakeholders who did not partake in the study may differ from those of participants who were willing and able to participate.

## Conclusion

In summary, all existing policy actions to reduce added sugar consumption in Australia targeted the food environment and behaviour change, were considered first-order changes and were mostly voluntary. Most policy actions proposed by informants also constituted first-order changes. Championing policies that preserve the existing system could reflect the influence of vested interests on the discourse around nutrition issues, the historical influence of reductionism on nutrition science and policy, and the perceived difficulty of modifying the current food system. Influences on nutrition policy actions to reduce added sugar consumption included industry power, fragmentation of stakeholders, government ideology and limited political will and public pressure. Few stakeholders had considered the unintended consequences of policy actions to reduce added sugar consumption, particularly in terms of NNS substitution. Pursuing nutrient-specific first-order changes to reduce added sugar intakes could lead to ‘regrettable’ substitutions and creates an opportunity cost for more comprehensive policy aimed at adjusting the broader food system.
